# Interactive visualisation for interpreting diagnostic test accuracy study results

**DOI:** 10.1136/ebmed-2017-110862

**Published:** 2018-01-24

**Authors:** Thomas R Fanshawe, Michael Power, Sara Graziadio, José M Ordóñez-Mena, John Simpson, Joy Allen

**Affiliations:** 1 Nuffield Department of Primary Care Health Sciences, University of Oxford, Oxford, UK; 2 NIHR Diagnostic Evidence Co-operative Newcastle, Newcastle upon Tyne Hospitals Foundation Trust, Newcastle upon Tyne, UK; 3 NIHR Diagnostic Evidence Co-Operative Newcastle, Newcastle University, Newcastle upon Tyne, UK

**Keywords:** decision threshold, diagnostic accuracy, probability, medical education, statistics and research methods

## Abstract

Information about the performance of diagnostic tests is typically presented in the form of measures of test accuracy such as sensitivity and specificity. These measures may be difficult to translate directly into decisions about patient treatment, for which information presented in the form of probabilities of disease after a positive or a negative test result may be more useful. These probabilities depend on the prevalence of the disease, which is likely to vary between populations. This article aims to clarify the relationship between pre-test (prevalence) and post-test probabilities of disease, and presents two free, online interactive tools to illustrate this relationship. These tools allow probabilities of disease to be compared with decision thresholds above and below which different treatment decisions may be indicated. They are intended to help those involved in communicating information about diagnostic test performance and are likely to be of benefit when teaching these concepts. A substantive example is presented using C reactive protein as a diagnostic marker for bacterial infection in the older adult population. The tools may also be useful for manufacturers of clinical tests in planning product development, for authors of test evaluation studies to improve reporting and for users of test evaluations to facilitate interpretation and application of the results.

## Background

Quantifying diagnostic accuracy is an important first step in assessing whether a new diagnostic device is suitable for implementation into clinical practice. Without initial evidence as to whether a device is able to improve diagnostic performance, it is difficult to justify larger studies to assess the impact on patient outcomes.

To many clinicians and researchers, statistical measures of diagnostic accuracy (which we refer to in this paper as ‘technical accuracy’) may appear counterintuitive and may not adequately reflect how a test result should influence decisions about the treatment of the patient.^1^ This difficulty arises because many test accuracy study results are expressed in terms of sensitivity and specificity rather than measures of ‘clinical accuracy’; that is, the probability that the patient has the disease or condition under consideration after receiving a positive or a negative test result.^2 3^

There is also evidence that many clinicians find it difficult to extract usable probabilistic information from diagnostic test accuracy results in the way that they are typically reported.^4 5^ However, there are conflicting opinions on the extent to which this depends on the type of information provided.^6^

The purpose of this article is twofold: to review the concepts of technical accuracy and clinical accuracy and highlight the measures of diagnostic performance that are particularly useful for statisticians, on the one hand, and patients and clinicians, on the other, and to demonstrate an interactive graphical interface to help medical educators and health professionals to teach, design and interpret the results of diagnostic accuracy studies.

### Example

Serum C reactive protein (CRP) is indicated as a marker of acute and chronic inflammation and bacterial infection and is widely used to assist in the diagnosis of these conditions.^7^ For illustration, we consider here the study of Liu *et al*,^8^ conducted in an older patient group (age >70 years). Defining elevated CRP levels as those exceeding 60 mg/L, the article reports the results in table 1 to show CRP test performance in relation to diagnosing bacterial infection, as assessed using a reference test based on clinical and microbiological criteria. The number of patients in each cell of the table is labelled as the number of true positive (TP), false positive (FP), false negative (FN) and true negative (TN) test results.

**Table 1:** Summary results table from a study of CRP and infection

		Reference test result		
		Definite, probable or possible infection	No infection	Total
CRP test result	Positive: elevated CRP (>60 mg/L)	TP=67	FP=6	73
	Negative: non-elevated CRP (<60 mg/L)	FN=16	TN=143	159
	Total	83	149	232

CRP, C reactive protein; FN, false negative; FP, false positive; TN, true negative; TP, true positive.

## Assessing diagnostic performance

Often, the diagnostic performance of the test is expressed using as summary statistics the sensitivity (proportion of infections correctly identified by the CRP test, TP/(TP+FN)=67/83=81%) and the specificity (proportion of non-infections correctly identified by the CRP test, TN/(FP+TN)=143/149=96%).^9^ Although widely used, these statistics do not by themselves enable the user to judge the probability that a patient who receives a particular CRP test result has infection. This probability depends additionally on the prevalence, or pre-test probability, of infection—how common bacterial infections are in the patient group under consideration. In this case, the estimated prevalence is 83/232=36%.

In the context of a single study, the relevant post-test probabilities, or ‘predictive values’, can be calculated directly. The data in table 1 enable us to estimate the positive predictive value (TP/(TP+FP)=67/73=92%) and the negative predictive value (TN/(FN +TN)=143/159=90%).

Disease prevalences may vary considerably between patient groups and care settings, even those in which the same diagnostic test is used. This has a substantial impact on predictive values. For example, a Swiss prospective cohort study of 218 patients aged >75 years found a lower prevalence of infection of 23% (50/218).^10^ However, provided the pre-test probability of infection is available, predictive values in the new population can be calculated on the assumption that the performance of the test remains the same. The prevalence of infection is likely to be a plausible estimate of the pre-test probability in the absence of other patient-specific information such as symptoms, signs or previous test results. 

Box **Calculation of post-test probabilities**

$$Positive\;Diagnostic\;Likelihood\;Ratio\left(DLR^{+}\right)=\frac{TP/\left(TP+FN\right)}{FP/\left(FP+TN\right)}=\frac{67/83}{6/149}=20.05$$

$$Post\text{-}test\;odds(+ve\;result)=DLR^{+}\times \frac{Prevalence}{1-Prevalence}=20.05\times \frac{50/218}{1-50/218}=5.97$$

$$Post\text{-}test\;probability\;(+ve\;result)=\frac{Post\text{-}test\;odds(+ve\;result)}{1+Post\text{-}test\;odds(+ve\;result)}=\frac{5.97}{6.97}=86\%$$

$$Negative\;Diagnostic\;Likelihood\;Ratio\;(DLR^{-})=\frac{FN/(TP+FN)}{TN/(FP+TN)}=\frac{16/83}{143/149}=0.201$$

$$Post\text{-}test\;odds\;(-ve\;result)=DLR^{-}\times \frac{Prevalence}{1-Prevalence}=0.201\times \frac{50/218}{1-50/218}=0.0598$$

$$Post\text{-}test\;probability\;(-ve\;result)=\frac{Post\text{-}test\;odds(-ve\;result)}{1+Post\text{-}test\;odds(-ve\;result)}=\frac{0.0598}{1.0598}=5.6\%$$

Using the 23% prevalence from Stucker *et al* 10 gives estimated probabilities of infection of 86% following a positive CRP test result and 5.6% following a negative test result. The Box provides details of the calculations, which use likelihood ratios^11^ estimated using the data from Liu *et al*.^8^ Both post-test probabilities are somewhat lower than those found in the setting described by Liu *et al*,^8^ which is a reflection of the reduced prevalence of infection in the Swiss population.

## Interactive graphical presentation

To help visualise and interpret the results of probability calculations when assessing diagnostic tests, we have created two free interactive tools, titled ‘Test Accuracy’ (https://micncltools.shinyapps.io/TestAccuracy)^12^ and ‘Clinical Accuracy and Utility’ (https://micncltools.shinyapps.io/ClinicalAccuracyAndUtility).^13^ These were developed using the RStudio application ‘Shiny’.^14^

**Figure 1:**
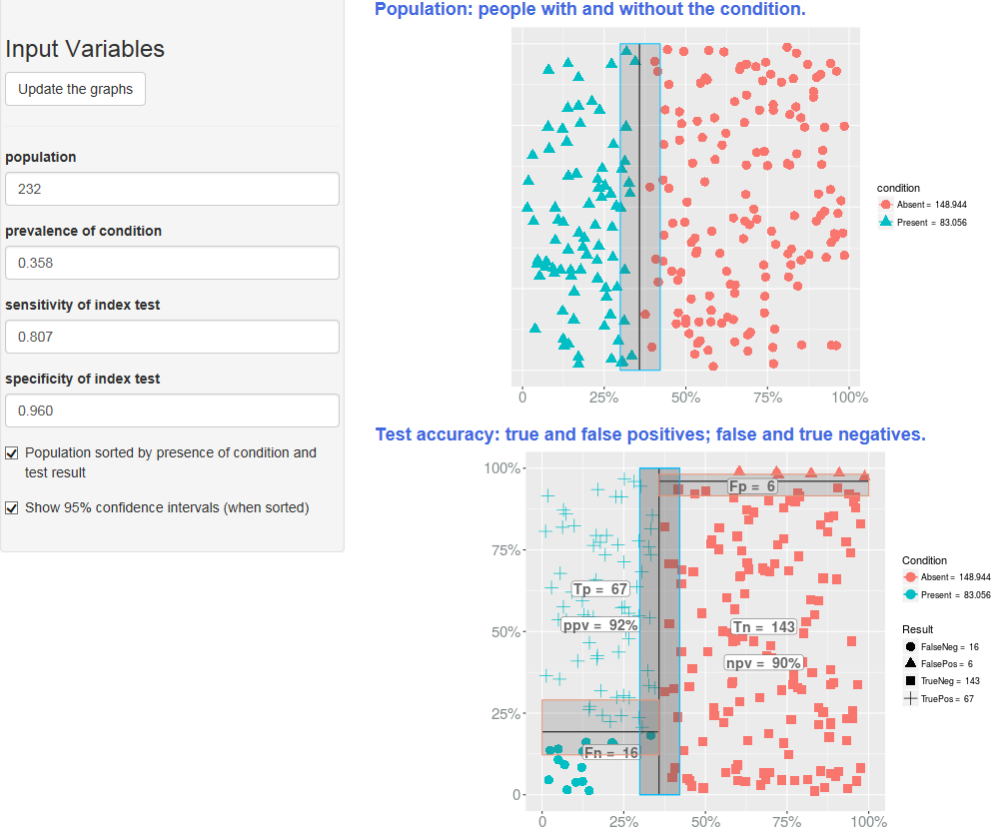
Screenshot from the ‘Test Accuracy’ tool, giving a graphical representation of parameters relating to diagnostic performance. FN, false negative; NPV, negative predictive value; PPV, positive predictive value; TN, true negative; TP, true positive.

The first of these provides a clear interface for illustrating measures of diagnostic technical accuracy, that is, sensitivity and specificity. It does so by showing the natural frequencies of TP, TN, FP and FN that would result for a given prevalence and sample size. The screenshot in figure 1 displays in graphical form the same information that is shown in table 1 for the study of CRP and infection.

**Figure 2:**
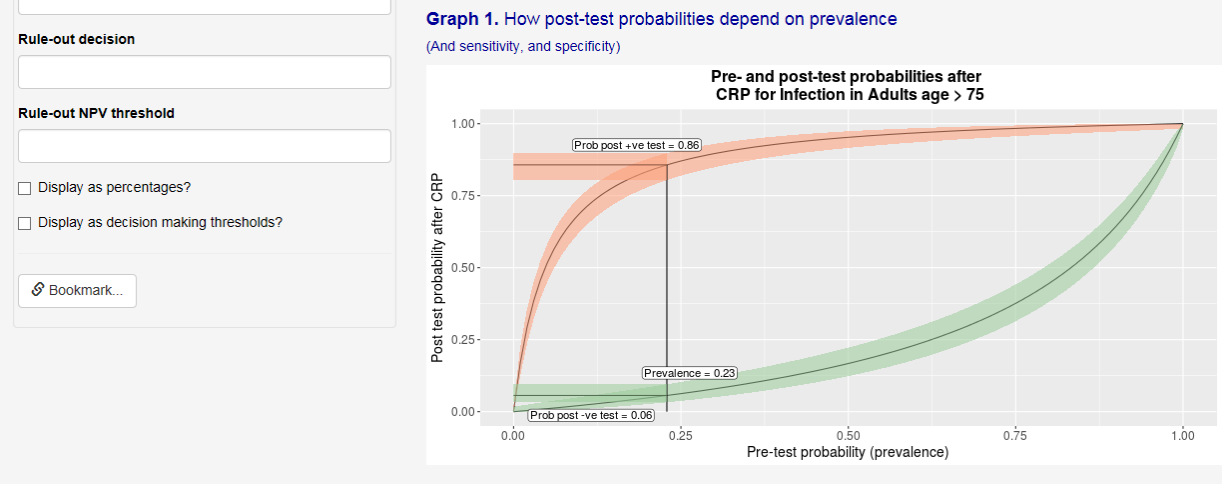
Screenshot from the ‘Clinical Accuracy and Utility’ tool, showing the relationship between disease prevalence (or pre-test probability) and post-test probability. CRP, C reactive protein.

The second tool is designed to help users to interpret pre-test and post-test probabilities of disease in relation to clinical decision thresholds.^15^ Figure 2 shows results based on the calculation described above, showing the hypothetical performance of the CRP test (the ‘Index Test’) in a population with 23% prevalence. Additionally, predictive probabilities are shown across the full range of possible prevalences from 0% to 100% to show the user the relationship between these two parameters. CIs are depicted as the coloured bands around each curve to aid communication of uncertainties associated with test accuracy on the resulting clinically relevant parameters.

The resulting predictive probabilities can easily be compared directly to rule-in or rule-out thresholds for clinical decision-making. In further options, these thresholds can be varied by the user, perhaps as a first step in performing a full decision curve analysis, in which decision-making is based on a trade-off between the consequences of FP and FN predictions.^16^

In practice, a range of decision thresholds has been proposed for CRP testing in different populations, as described in systematic reviews on the subject.^7 17^ For the purpose of illustration, suppose that a policy recommendation suggests that a particular treatment be initiated if the post-test probability of treatment exceeded 90%. Using the interactive tools, the user can change the available parameters to see the effect of improved or reduced performance of the test in a different setting, or the different prevalence of disease that might better reflect the characteristics of a new population. Varying the prevalence of disease (figure 2) shows that, given the performance of the diagnostic test, this threshold would be exceeded for individuals who receive a positive test result only in populations for which the disease prevalence is above around 30%. The threshold would therefore not be exceeded in the lower prevalence setting of the Swiss study described above.

These tools are intended to help those involved in communicating information about diagnostic test performance and are likely to be of benefit when teaching these concepts. They may also be useful for manufacturers of clinical tests in planning product development, for authors of test evaluation studies to improve reporting and for users of test evaluations to facilitate interpretation and application of the results. Example scenarios include those in which predictive values are not provided directly, but can be inferred from sensitivity, specificity and prevalence information, and situations in which the prevalence of the condition varies. They could also be useful for authors of systematic reviews of diagnostic test accuracy studies to derive predictive values from sensitivity and specificity values. They have value in designing new studies, for which preliminary estimates of predictive values and their CIs are useful in helping to choose appropriate and ethical sample sizes. The tool quickly allows users to assess the impact of different sample size and prevalence assumptions on CIs, which can be compared directly against a decision-making threshold.

## Conclusion

In summary, the clinical accuracy of diagnostic tests, as expressed by post-test probabilities, may be used to guide treatment decisions. These probabilities may vary across different populations. We have created two free, interactive tools to help to visualise these concepts. Future work may include extending these tools to incorporate diagnostic results based on continuous measurements.
